# *Atractylodes chinensis* Water Extract Ameliorates Obesity via Promotion of the SIRT1/AMPK Expression in High-Fat Diet-Induced Obese Mice

**DOI:** 10.3390/nu13092992

**Published:** 2021-08-27

**Authors:** Yea-Jin Park, Min-gyu Seo, Divina C. Cominguez, Insik Han, Hyo-Jin An

**Affiliations:** 1 Department of Pharmacology, College of Korean Medicine, Sangji University, Wonju 26339, Korea; wer0928@hanmail.net (Y.-J.P.); namusup00@naver.com (M.-g.S.); divina_0406@yahoo.com (D.C.C.); 2 Department of Internal Medicine of Korean Medicine, Oriental Medicine Hospital of Sangji University, Wonju 26338, Korea; lufice85@sangji.ac.kr

**Keywords:** *Atractylodes chinensis*, AMPK, SIRT1, lipogenesis, fatty acid oxidation

## Abstract

Obesity remains a continuing global health concern, as it is associated with an increased risk of many chronic diseases. *Atractylodes chinensis* Koidz. (Ac) is traditionally used in the treatment of inflammatory diseases, such as arthritis, hepatitis, and gastric ulcers. Despite the diverse pharmacological activities of Ac, scientific evidence for the use of Ac in obesity is still limited. Therefore, the present study aimed to determine the anti-obesity effects of Ac. C57BL/6N mice were divided into five groups as follows: chow diet group (CON), 45% HFD group, HFD + oral administration of orlistat group, and HFD + oral administration of Ac groups. RT-PCR and western blotting were used to examine the expression of molecules relating to obesity progression. Ac-administered mice showed dramatically decreased body weight and weight gain compared to the high-fat diet (HFD)-fed mice. In addition, Ac administration attenuated the protein expression levels of adipogenic transcription factors in the white adipose tissue (WAT) and livers of HFD-fed mice. Furthermore, Ac administration declined the expression levels of lipogenic genes, while enhancing those of the fatty acid oxidation genes in the WAT of HFD-fed mice. Importantly, Ac administration highly upregulated the AMP-activated kinase (AMPK) and sirtuin 1 (SIRT1) expression levels in WAT of the HFD-induced obese mouse model. Our results provide evidence that Ac can effectively ameliorate weight gain and adipose tissue expansion.

## 1. Introduction

Obesity, defined as a body mass index (BMI) greater than or equal to 30.0 kg/m^2^, remains a continuing global health concern, as it is linked with an increased risk of many chronic diseases, such as type 2 diabetes, cardiovascular disease, hypertension, and several cancers [1]. White adipose tissue (WAT) performs complex metabolic functions under physiological conditions, but during obesity, WAT is extremely dysfunctional and fails to appropriately expand to store surplus energy; consequently, it causes ectopic fat deposition in several tissues that modulate metabolic homeostasis [2]. In this regard, it could be very useful to design strategies that decrease lipogenesis and increase lipid mobilization and oxidation in the treatment of obesity and related disorders [3]. In addition, it was reported that visceral adipose tissue, compared with subcutaneous adipose tissue, includes plenty of inflammatory and immune cells, is more metabolically active, more sensitive to lipolysis, and has greater insulin resistance [4]. Therefore, the regulation of the expansion of visceral adipose tissue via decreasing lipogenesis and promoting lipid oxidation can be a potential therapeutic avenue in obesity and related metabolic dysfunction.

Sirtuins (SIRTs) are characterized by NAD^+^-dependent deacetylase activity [5]. Humans have seven SIRTs, SIRT1–SIRT7, and SIRT1 is the most studied member of this family and plays a key role in various processes ranging from cell cycle modulation to energy homeostasis [6]. SIRT1 has important functions in glucose and fat metabolism in several tissues, including the adipose tissue, liver, and pancreas [7,8,9,10,11,12], and it has been reported that pharmacological activation of SIRT1 may attenuate metabolic dysfunction linked with obesity and other metabolic dysfunctions [6]. Indeed, resveratrol, a naturally occurring SIRT1 agonist, was shown to alleviate diet-induced obesity and insulin resistance in mice [13]. SIRT1 plays an important role in tissues linked with fat metabolism, including visceral fat; therefore, it can be a master target gene for management of obesity and related diseases [14].

Many studies have shown that natural products, including medicinal plants, have a variety of pharmacological and biological properties and show potential as proper candidates for the treatment of various diseases, such as psychiatric disorders [15], inflammatory pain disorder [16], and cancer [17]. The genus *Atractylodes* (Asteraceae) is used as a medicinal herb in East Asia [18]. Atractylodes rhizoma is ubiquitously used to treat rheumatic diseases, influenza, night blindness, and several other types of digestive disorders [19]. The dried rhizomes have been classified into two types: Baizhu and Cangzhu. Among them, the rhizome of *Atractylodes chinensis* Koidz. (Ac) is classified as Changzhu [20]. Ac is traditionally used in the treatment of inflammatory diseases, such as arthritis, hepatitis, and gastric ulcers [20], as well as to invigorate the spleen and eliminate dampness [21]. Previous study revealed that phlegm-dampness constitution is one of the high-risk factors of metabolic diseases, containing obesity, and studies with traditional Chinese medicine have significant importance for the prevention of associated diseases [22]. Despite the diverse pharmacological activities and potential for anti-obesity effects of Ac, scientific evidence for the use of Ac in obesity is still insufficient. Therefore, we hypothesized that Ac may prevent against development of obesity by regulating lipid metabolism through activation of SIRT1 in visceral adipose tissue.

## 2. Materials and Methods

### 2.1. Preparation of Ac

Dried roots of Ac were purchased from Nanum Herb (Yeongchen, Gyeongbuk, Korea). Extract was prepared from 100 g of dried roots of Ac with hot water (1 L). Supernatants of the extraction were collected, filtered, and freeze-dried, and the yield was calculated at 30.18% (30.18 g per 100 g of Ac). The powder was dissolved in distilled water for in vivo analysis.

### 2.2. HFD-Induced Obesity Mouse Model

All experimental procedures were approved by the Ethical Committee for Animal Care and Use of Laboratory Animals of Sangji University (reg.no. 2018-28). Briefly, thirty male C57BL/6N mice (20 ± 2 g) at 8 weeks of age were used in these animal experiments (n = 6 per group). Mice were housed in a 12 h light/dark cycle, 22 ± 2 °C, and 55% ± 9% humidity. After a week of acclimation, mice were fed either a chow diet (CON) or 45% HFD (D-12451, Research Diets (New Brunswick, NJ, USA)). For drug treatment, orlistat (20 mg/kg) or Ac (25 or 50 mg/kg) were administrated to mice via oral gavage for 8 weeks. The orlistat was obtained from Tokyo Chemical Inc. (Tokyo, Japan). The compositions of HFD and chow diet are presented in Table 1. Mice had free access to water and diet. Body weight and food intake were monitored weekly. At the end of the experiments, all animals were euthanized by Zoletil 50 (20 mg/kg, i.p.) and cervical dislocation. Liver and adipose tissues were taken and rapidly stored at −80 °C.

### 2.3. Blood Metabolite Profile Analysis

Serum triglyceride (TG), total cholesterol (TC), alanine aminotransferase (ALT), and blood urea nitrogen (BUN) concentrations were measured using enzymatic methods with commercially available kits (Asan Pharmaceutical. Co. Ltd., Seoul, Korea). All biochemical assays were performed according to the manufacturer’s instructions.

### 2.4. Histological Analysis

Liver and eWAT were fixed in 10% buffered formalin and then embedded in paraffin. The 5 µm thick sections were deparaffinized, rehydrated, and stained with hematoxylin and eosin for histological examination. The images were acquired using an Olympus SZX10 microscope (Tokyo, Japan).

### 2.5. Western Blot Analysis

The liver and epididymal WAT were homogenized and the protein expression was conducted as described [23]. Membranes were incubated overnight with specific primary antibody (Table 2) and then with horseradish peroxidase-conjugated secondary antibody (Jackson ImmunoResearch Laboratories, Inc. (West Grove, PA, USA)) for 2 h. The blots were then visualized by enhanced chemiluminescence using AmershamTM Imager 680 (GE Healthcare Bio-Sciences AB, Uppsala, Sweden).

### 2.6. Quantitative Reverse-Transcription Polymerase Chain Reaction (qRT-PCR) Analysis

The liver and epididymal WAT were homogenized and the mRNA levels was conducted using a Step One Plus Real-time PCR system (Applied Biosystems, Thermo Fisher Scientific, Inc., Waltham, MA, USA) as described [23]. GAPDH was used as an internal control. The sequences of the mouse oligonucleotide primers (Bioneer Corporation (Daejeon, Korea)) are shown in Table 3.

### 2.7. Statistical Analysis

The results are given as the mean ± standard deviation. Statistical analysis was conducted using SPSS (version 19.0; International Business Machines, Armonk, NY, USA). Statistical significance was performed using analysis of variance and Dunnett’s post hoc test. Statistical significance was set at *p* < 0.05.

## 3. Results

### 3.1. Reduced Body Weight, Serum Triglyceride, and Total Cholesterol Levels in Ac-Treated Obese Mice

**Figure 1:**
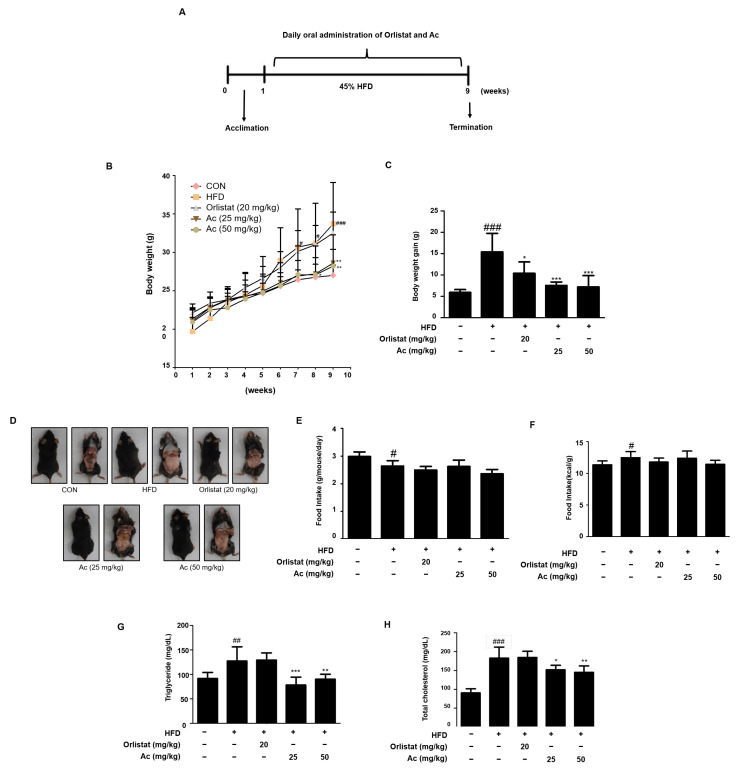
Effect of Ac on body weight, food intake, and TG and TC levels in HFD-induced obese mice. (**A**) A flow chart showing the experimental procedure. (**B**) Body weight. (**C**) Body weight gain. (**D**) Abdominal fat content. (**E**,**F**) Food intake. The levels of serum (**G**) TG and (**H**) TC. Data are expressed as the mean ± SD # *p* < 0.05, ## *p* < 0.01, and ### *p* < 0.001 vs. chow diet-fed group; * *p* < 0.05, ** *p* < 0.01, and *** *p* < 0.001 vs. only HFD-fed group.

To investigate whether Ac administration affects obesity, an HFD-induced obese mouse model was used (Figure 1A). HFD remarkably increased the body weight and weight gain of the mice. However, the Ac-administered groups showed significantly decreased body weight and weight gain compared to the HFD group (Figure 1B,C). Macroscopic analysis also revealed reduced abnormal fat content in Ac-treated mice compared to HFD-fed mice (Figure 1D). Importantly, the inhibitory effect of Ac was not attributable to reduced food intake (Figure 1E,F). Additionally, the serum levels of TG and TC were highly elevated by HFD, but Ac administration significantly reduced serum levels of TG and TC (Figure 1G,H). Furthermore, liver and kidney toxicity was not observed with Ac treatment (Table 4).

### 3.2. Reduced Adipose Tissue Expansion and Adipocyte Differentiation in eWAT of Ac-Treated Obese Mice

**Figure 2:**
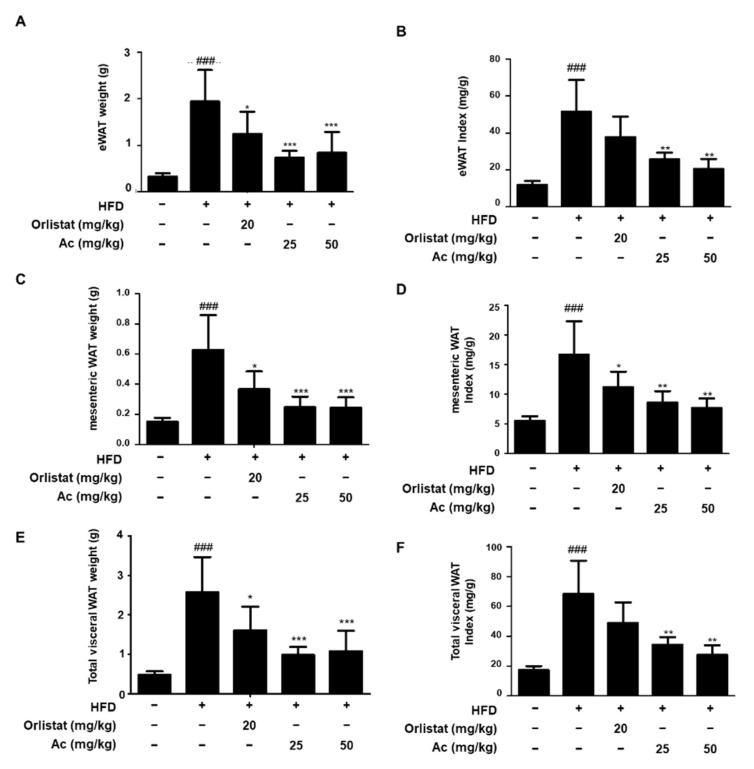
Effect of Ac on the weights of WAT in HFD-induced mice. (**A**) The eWAT weight; (**B**) eWAT index (mg/body weight); (**C**) mesenteric WAT weight; (**D**) mesenteric WAT index (mg/body weight); (**E**) total visceral WAT weight; (**F**) total visceral WAT index (mg/body weight). Index is the value of WAT weight divided by mouse body weight. Data are expressed as the mean ± SD ### *p* < 0.001 vs. chow diet-fed group; * *p* < 0.05, ** *p* < 0.01, and *** *p* < 0.001 vs. only HFD-fed group. Abbreviations: WAT, white adipose tissue.

**Figure 3:**
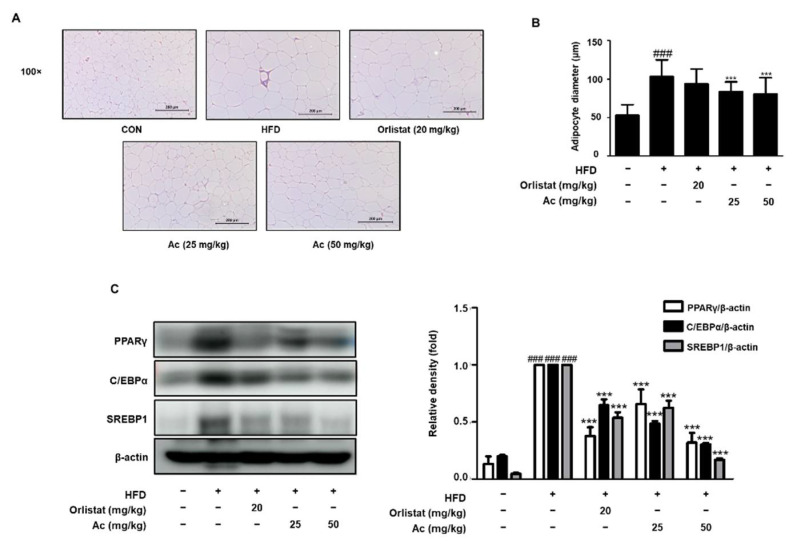
Effect of Ac on the adipose tissue expansion and adipogenic transcription factors in eWAT of HFD-induced mice. (**A**) Representative images stained with H&E in eWAT. Scale bar is 200 µm. (**B**) The average diameter of adipocytes in eWAT. (**C**) The protein level of PPARγ, C/EBPα, and SREBP1 in eWAT analyzed by western blot analysis. Densitometric analysis was performed using ImageJ v1.50i. Data are expressed as the mean ± SD of three independent experiments. ### *p* < 0.001 vs. chow diet-fed group; *** *p* < 0.001 vs. only HFD-fed group.

Next, in order to assess whether Ac reduces visceral WAT expansion, we checked the effect of Ac on the WAT mass. The analysis of adipose tissue mass revealed significantly lower WAT (epididymal, mesenteric, and total visceral) weights in the Ac-treated groups than in the HFD group (Figure 2A,C,E). Furthermore, the WAT indexes, WAT weight divided by mouse body weight, were also significantly decreased in Ac-treated groups (Figure 2B,D,F). These data show that Ac administration elicits beneficial effects on the reduction of weight gain and WAT mass. Hematoxylin and eosin staining showed that Ac administration effectively decreased adipocyte size and diameter compared to the HFD group (Figure 3A,B). Next, to elucidate the mechanisms contributing to epididymal WAT (eWAT) mass reduction in Ac-treated mice, we analyzed the expression of adipogenic transcription factors in eWAT. Western blot analysis revealed significantly lower protein expression of peroxisome proliferator-activated receptor γ (PPARγ), CCAAT/enhancer-binding protein α (C/EBPα), and sterol regulatory element-binding protein (SREBP1) in Ac-treated groups than in the HFD group (Figure 3C).

### 3.3. Reduced Lipid Metabolism in eWAT of Ac-Treated Obese Mice

**Figure 4:**
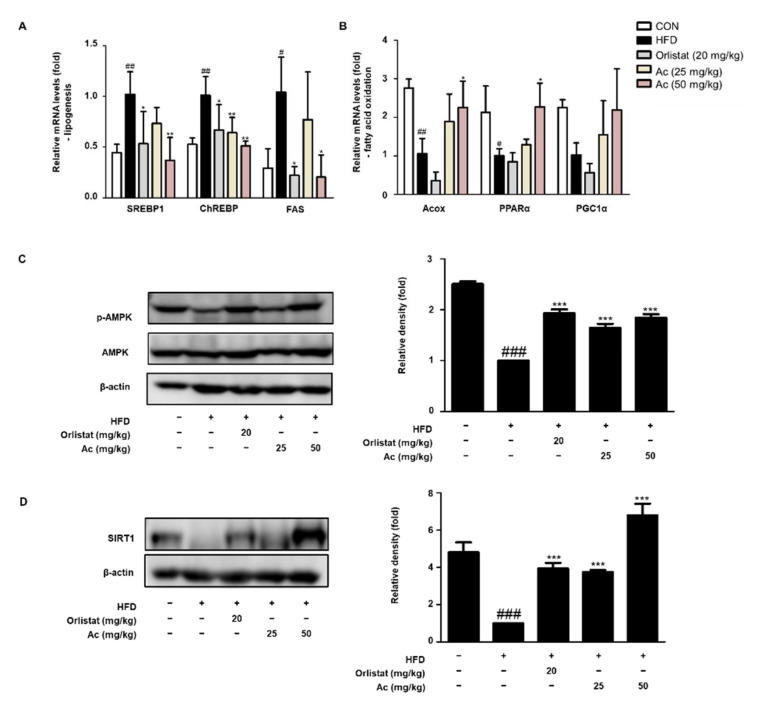
Effect of Ac on the lipogenesis and fatty acid oxidation markers in eWAT tissue of HFD-induced obese mice. The mRNA levels of (**A**) SREBP1, ChREBP, FAS, (**B**) Acox, PPARα, and PGC1α in eWAT assessed by qRT-PCR. The protein levels of (**C**) p-AMPK, AMPK, and (**D**) SIRT1 analyzed by western blot analysis. Densitometric analysis was performed using ImageJ v1.50i. Data are expressed as the mean ± SD of three independent experiments. # *p* < 0.05, ## *p* < 0.01, and ### *p* < 0.001 vs. chow diet-fed group; * *p* < 0.05, ** *p* < 0.01, and *** *p* < 0.001 vs. only HFD-fed group.

Increased lipogenesis and decreased β-oxidation lead to lipid accumulation in adipose cells [24]. To distinguish the molecular mechanisms of Ac on the reduction of eWAT mass, lipogenesis and fatty acid oxidation markers in eWAT were investigated by real-time PCR analysis. As for fatty acid biosynthesis, the mRNA levels of SREBP1, carbohydrate response element-binding protein (ChREBP), and fatty acid synthase (FAS) were higher in the HFD group than in the CON group, whereas these markers were markedly diminished in the Ac-administered groups compared to the HFD group (Figure 4A). As for fatty acid oxidation, acyl-CoA oxidase (Acox) and peroxisome proliferator-activated receptor α (PPARα) mRNA levels were lower in the HFD group than in the CON group. However, these markers were strongly elevated in the Ac-administered mice compared to the HFD group (Figure 4B). Additionally, similar trends were observed for the PPARγ coactivator 1α (PGC1α) mRNA levels (Figure 4B). Furthermore, AMP-activated kinase (AMPK) phosphorylation was significantly downregulated in the HFD group compared to that in the CON group, whereas Ac administration significantly upregulated p-AMPK expression (Figure 4C). Next, we analyzed SIRT1 expression to examine whether the changes in expression of adipogenic, lipigenic, and fatty acid oxidation-related genes after Ac administration were regulated by SIRT1. Notably, SIRT1 protein expression was significantly enhanced in the Ac-administered groups compared to that in the HFD group (Figure 4D).

### 3.4. Reduced Steatosis and Adipogenesis in the Liver of Ac-Treated Obese Mice

**Figure 5:**
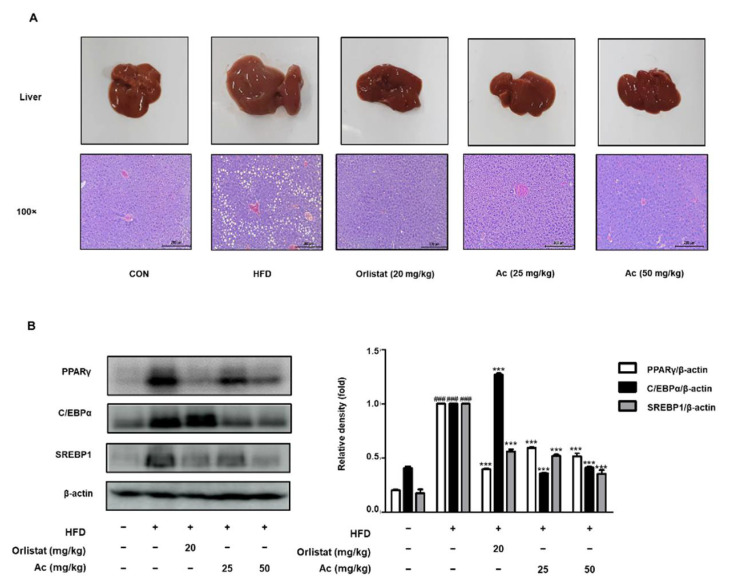
Effect of Ac on the adipogenesis in the liver tissue of HFD-induced obese mice. (**A**) Macroscopic analysis and representative images stained with H&E in liver tissue. Scale bar is 200 µm. (**B**) the protein level of PPARγ, C/EBPα, and SREBP1 in the liver analyzed by western blot analysis. Data are expressed as the mean ± SD of three independent experiments. ### *p* < 0.001 vs. chow diet-fed group; *** *p* < 0.001 vs. only HFD-fed group.

Abnormal WAT expansion causes ectopic fat deposition in other tissues, including the liver. Macroscopic analysis and hematoxylin and eosin staining of the liver sections revealed that a number of lipid droplets accumulated in the liver tissue in the HFD group compared to the CON group. However, Ac alleviated these pathological changes after being administered for 8 weeks of treatment (Figure 5A). Similar to eWAT, HFD increased the adipogenic protein expression of PPARγ, C/EBPα, and SREBP1 compared to the CON group, whereas Ac administration significantly downregulated PPARγ, C/EBPα, and SREBP1 compared to the HFD group (Figure 5B).

## 4. Discussion

Obesity is characterized by the expansion of the adipose tissue. Obesity is associated with metabolic disorders because visceral adipose tissue contains various inflammatory and immune cells [4]; it shows that there is a relationship between obesity and inflammation. A previous report revealed that the rhizome of Ac has anti-inflammatory effects via suppression of the Akt/IκB/NF-κB signaling [20], therefore, we hypothesized that Ac may prevent against development of obesity by affecting visceral adipose tissue. WAT expansion is achieved through hypertrophy or hyperplasia, and the latter process involves adipogenesis [2]. Adipogenesis is driven by an intrinsic network of transcription factors, PPARγ and C/EBPα, at the epicenter [2]. Moreover, SREBP1c, a transcription factor, appears to contribute to PPARγ expression and is regulated directly by C/EBP factors during adipogenesis [25]. In this study, we observed that Ac administration attenuated the protein expression of PPARγ, C/EBPα, and SREBP1 in the WAT and liver of HFD-induced obese mice (Figure 3C and Figure 5B). These results attest to the potential of Ac administration as a white adipose tissue reducer and liver steatosis attenuator via the inhibition of adipocyte differentiation.

We hypothesize that the decreased white adipose tissue mass might also be related to the modulation of lipogenesis and fatty acid oxidation. SREBP1 also controls lipid biosynthesis and adipogenesis by modulating the expression of several enzymes required for triacylglycerol, cholesterol, fatty acid, and phospholipid synthesis [26]. The physiological role of SREBP1 in response to nutrition was initially proposed for the modulation of lipogenic genes, such as FAS, in adipose tissue and liver [27]. Furthermore, ChREBP controls fatty acid synthesis, elongation, and desaturation by promoting FAS and ACC1 [28]. Our results showed that Ac administration diminished the expression of the lipogenic genes SREBP1, Chrebp, and FAS in the eWAT of the HFD-induced obese mouse model (Figure 4B).

As a transcription factor, PGC-1α induces the levels of several genes of the citric acid cycle and the mitochondrial fatty acid oxidation pathway [29], and can bind to targets including PPARα, PPARβ/δ, and PPARγ, which regulate the levels of mitochondrial genes and indirectly contribute to transport and utilization of fatty acids [29]. Acox initiates peroxisomal β-oxidation, catalyzing the desaturation of acyl-CoA to 2-trans-enoyl-CoA. Acox1 preferentially catabolizes straight-chain fatty acids, but the enzymes Acox2 and Acox3 are thought to use branched-chain fatty acids and intermediates linked with bile acid synthesis as substrates [30]. Our results showed that Ac administration promoted the levels of fatty acid oxidation-related genes, Acox, PPARα, and PGC-1α, in the eWAT of the HFD-induced obese mice (Figure 4B). Overall, we found that Ac administration reduced lipogenesis by decreasing the expression of SREBP1, Chrebp, and FAS, and promoted fatty acid oxidation by increasing Acox, PPARα, and PGC-1α; our results suggest that Ac administration attenuates adipogenesis and lipogenesis and promotes fatty acid oxidation, consequently preventing WAT expansion.

The SIRT1/AMPK pathway is a key regulator of the energy balance. When AMPK is activated, it initiates metabolic and genetic events that restore ATP levels by promoting processes that generate ATP, including fatty acid oxidation, and suppressing others that consume ATP but are not acutely required for survival, including TG and protein synthesis and cell proliferation [31]. Sirtuins, such as AMPK, serve as bona fide cell energy sensors; their activation responds to alternations in the availability of the metabolic cofactor NAD^+^ and the involved intermediates, NADH and nicotinamide, causing a state of caloric restriction or starvation, which elevates the NAD^+^/NADH or NAD^+^/nicotinamide ratios, leading to enhanced SIRT1 content and activation in other tissues [32,33,34]. Decades of evidence have shown that the partnership between AMPK and SIRT1 has numerous potential implications for the pathogenesis and therapy of disorders linked with metabolic dysfunction [31]. SIRT1 stimulates AMPK by deacetylating its upstream activator, which in turn causes an increase in NAD^+^ levels, consequently enhancing deacetylation/activation of other SIRT1 targets linked with fatty acid oxidation, such as PGC-1α [3]. Several observations have also suggested that activation of SIRT1/AMPK signaling may offer critical pharmacological benefits in the treatment of dyslipidemia, obesity, and metabolic syndrome [35,36,37,38], and we found that Ac administration highly enhanced the AMPK and SIRT1 expression levels in the eWAT of the HFD-induced obese mouse model (Figure 4C,D). These findings provide evidence that Ac can effectively reduce the WAT expansion via the regulation of AMPK and SIRT1 expression levels, thereby preventing weight gain.

It was reported that SIRT1 regulates inflammatory pathway activation as well as insulin signaling in adipocytes [39]. Furthermore, SIRT1 blocks infiltration of macrophages and promotes M2 macrophage polarization within adipocytes, thereby protecting the onset and progression of metabolic disorders [40]; this implies that Ac also may attenuate inflammatory responses in adipose tissue via SIRT1 activation, though further study on this is necessary. HFD consumption induces alterations in the gut microbial profile as well as decreased diversity, which promotes obesity and consequently leads to metabolic diseases [41]. In this respect, Ac may also indirectly act on HFD-induced gut microbial changes in obesity, and subsequent can affect visceral adipose tissue; therefore, we are going to assess gut microbiota and adipose tissue inflammation to clarify the regulatory effects of Ac on obesity. Although additionally studies (even clinical) are needed to further understand the mechanisms of Ac on obesity, our results suggest that Ac could be a candidate for the prevention of obesity.

## 5. Conclusions

Taken together, our findings suggest that Ac attenuates WAT expansion by reducing adipogenesis and lipogenesis and promoting the fatty acid oxidation via the regulation of AMPK and SIRT1 expression levels.

## Figures and Tables

**Table 1:** The composition of HFD and chow diet.

Content (kcal%)	Diets	
	HFD	Chow Diet
Fat %	45	5
Protein %	20	18
Carbohydrate %	35	75.9
Energy (kcal/kg)	4730	3811
Cholesterol (mg/kg)	196.5	-
Protein ingredients	Casein, L-Cystein	-
Fat ingredients	Soybean oil, Lard	-
Carbohydrate ingredients	Corn Starch, Maltodextrin, Sucrose	-

**Table 2:** Antibodies for Western blotting.

Antibody	Dilution	Source	Vendor	Catalog No.
PPARγ	1:2000	Mouse	Santa Cruz Biotechnology	sc-7273
SREBP1	1:2000	Mouse	Santa Cruz Biotechnology	sc-13551
C/EBPα	1:1000	Mouse	Santa Cruz Biotechnology	sc-365318
p-AMPK	1:2000	Rabbit	Cell Signaling Technology	#2531
AMPK	1:2000	Rabbit	Cell Signaling Technology	#2532
SIRT1	1:1000	Mouse	Santa Cruz Biotechnology	Sc-74465
β-actin	1:2500	Mouse	Santa Cruz Biotechnology	sc-81178

**Table 3:** Real-time polymerase chain reaction (PCR) primer sequences.

Gene	Forward (5′-3′)	Reverse (3′-5′)
SREBP1	ATCGCAAACAAGCTGACCTG	AGATCCAGGTTTGAGGTGGG
ChREBP	CACTCAGGGAATACAGCGCTAC	ATCTTGGTCTTAGGGTCTTCAGG
FAS	AGGGGTCGACCTGGTCCTCA	GCCATGCCCAGAGGGTGGTT
Acox	TAACTTCCTCACTCGAAGCCA	AGTTCCATGACCCATCTCTGTC
PPARα	CAGGAGAGCAGGGATTTGCA	CCTACGCTCAGCCCTCTTCAT
PGC1α	TATGGAGTGACATAGAGTGTGCT	CCACTTCAATCCACCCAGAAAG
GAPDH	GACGGCCGCATCTTCTTGT	CACACCGACCTTCACCATTTT

**Table 4:** The liver and kidney toxicity in the serum of Ac-treated obese mice.

	Parameters	
Groups	ALT (U/L)	BUN (mg/dL)
CON	28.40 ± 6.53	22.05 ± 2.46
HFD	37.36 ± 5.44	22.18 ± 1.76
Orlistat (20 mg/kg)	33.99 ± 5.12	22.53 ± 1.29
Ac (25 mg/kg)	30.99 ± 7.24	21.79 ± 2.89
Ac (50 mg/kg)	30.36 ± 3.50	18.41 ± 1.08

The values are represented as mean ± SD (*n* = 4). There were no statistically significant differences in the parameters from all groups. Abbreviations: ALT, alanine aminotransferase; Ac, *Atractylodes chinensis* Koidz; BUN, blood urea nitrogen; CON, chow diet; HFD, high-fat diet.

## Data Availability

The datasets used and/or analyzed in the present study are available from the corresponding authors upon reasonable request.
